# Linking path and filament persistence lengths of microtubules gliding over kinesin

**DOI:** 10.1038/s41598-022-06941-x

**Published:** 2022-02-23

**Authors:** May Sweet, Samuel Macharia Kang’iri, Takahiro Nitta

**Affiliations:** Applied Physics Course, Faculty of Engineering, Gifu University, Gifu, 501-1193 Japan

**Keywords:** Motility, Nanobiotechnology, Nanoscale devices, Molecular machines and motors

## Abstract

Microtubules and kinesin motor proteins are involved in intracellular transports in living cells. Such intracellular material transport systems can be reconstructed for utilisation in synthetic environments, and they are called molecular shuttles driven by kinesin motors. The performance of the molecular shuttles depends on the nature of their trajectories, which can be characterized by the path persistence length of microtubules. It has been theoretically predicted that the path persistence length should be equal to the filament persistence length of the microtubules, where the filament persistence length is a measure of microtubule flexural stiffness. However, previous experiments have shown that there is a significant discrepancy between the path and filament persistence lengths. Here, we showed how this discrepancy arises by using computer simulation. By simulating molecular shuttle movements under external forces, the discrepancy between the path and filament persistence lengths was reproduced as observed in experiments. Our close investigations of molecular shuttle movements revealed that the part of the microtubules bent due to the external force was extended more than it was assumed in the theory. By considering the extended length, we could elucidate the discrepancy. The insights obtained here are expected to lead to better control of molecular shuttle movements.

## Introduction

**Figure 1:**
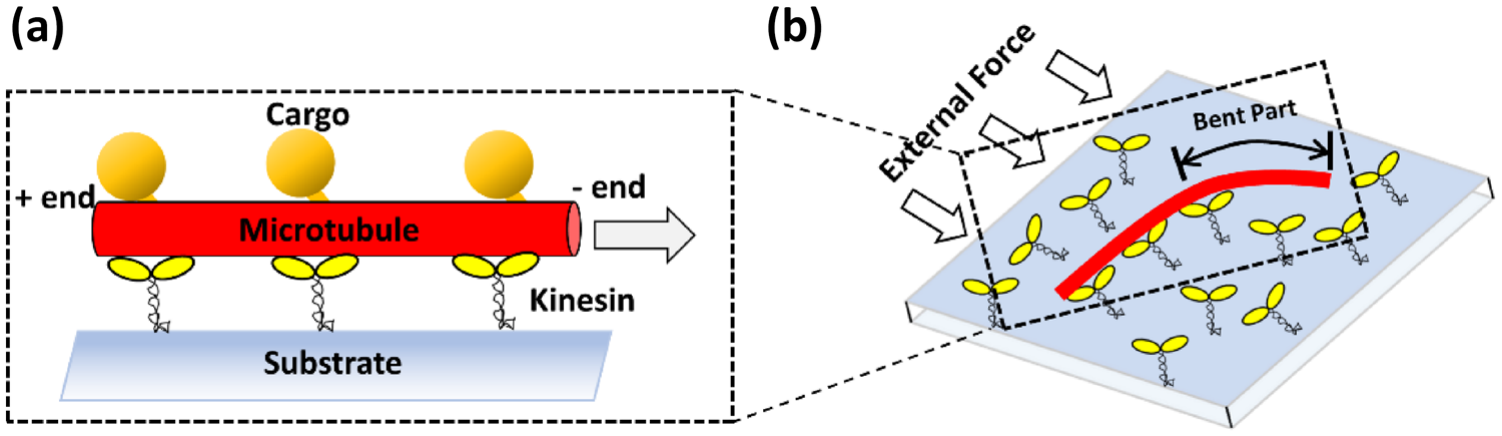
Schematic drawings of (**a**) a microtubule-based molecular shuttle (MS) powered by kinesin motors and (**b**) movements of MSs under an externally applied force field.

Cytoskeletal filaments and the associated motor proteins, such as actin filaments and myosin, or microtubules (MTs) and kinesins, have essential roles in living cells but can also be applied in synthetic environments^1–5^. Their small size and highly robust and efficient motilities make them ideal power sources to drive microscopic devices for sensing^6^, computation^7^, optics^8^, and robotics^9,10^. Molecular shuttles (MSs) powered by motor proteins^11,12^ are a well-studied form that utilizes the cytoskeletal filaments and the associated motor proteins. MSs are made of cytoskeletal filaments gliding over surfaces covered with motor proteins (Fig. 1a), and they are used for delivering cargo along a predefined track on biosensors^13–15^ and for parallel computation^7^.

Movements of MSs are crucial for the overall device performance. MSs take trajectories with directional fluctuations. The degree of the fluctuation is characterized by the path persistence length (${L}_{p, path}$). The path persistence length has been experimentally measured to be 0.1–0.5 mm for microtubule-based MSs gliding over kinesin motors^16–19^ and 0.01 mm for actin filament-based MSs gliding over myosin motors^20,21^.

The path persistence length is theoretically predicted to be equal to the filament persistence length (${L}_{p,filament}$)^22^, where the latter is a measure of the bending stiffness of the filament. This prediction is deduced as follows: during translocation, as cytoskeletal filaments are propelled by the associated motor proteins, the free suspended part of the translating cytoskeletal filaments thermally fluctuates. Assuming that motor proteins binding to cytoskeletal filaments act as rigid anchors, once the leading tip of the cytoskeletal filament binds with a motor protein, the conformation of the cytoskeletal filament “freezes”. Thus, directional fluctuation along a trajectory of a cytoskeletal filament reflects that of the conformation of the filament itself. Hence, the path persistence length is predicted to be equal to the filament persistence length. However, the filament persistence length of microtubules has been reported to be 1–5 mm^23^. Thus, there is a tenfold difference between path and filament persistence lengths of microtubules, contradicting the theory.

A possible explanation for this large discrepancy is the length-dependent flexural rigidity of MTs^24,25^. The length-dependent flexural rigidity of MTs is a consequence of anisotropic interactions between tubulins and leads to low flexural rigidity for short MTs. Indeed, previous studies showed that, based on the length-dependent flexural rigidity of MTs, the discrepancy could be accounted for^17,18,26^. However, it still remains unclear if the flexural rigidity of MTs really depends on the length^27^. In addition, the flexural rigidity of MTs depends on their preparation and growth conditions^28^, making it complicated to compare ${L}_{p,path}$ and ${L}_{p,filament}$ of MTs of separate experiments.

In this study, we report how the discrepancy between ${L}_{p,path}$ and ${L}_{p,filament}$ of MTs arises using computer simulation. A simulation study has distinct advantages over an experimental one. First, since in the simulation, ${L}_{p,filament}$ is a preset parameter, comparison between ${L}_{p,path}$ and ${L}_{p,filament}$ and its interpretation are straightforward and avoid complexities associated with MT preparations in experiments. Second, the precise locations of bound kinesins are easily obtained in the simulation while in experiments such information is only available under limited conditions^29^. Using these advantages, we test a hypothesis that treating kinesins as flexible anchors may alter the path persistence length with a simulation of MTs gliding over kinesins under external forces. While the path persistence length can be calculated from trajectories without external forces, the use of external forces enables explicit calculation of the length of the fluctuating part of MTs as that of the bent part because the front part of the MT is either freely suspended or loosely anchored by kinesin motors. In the following, we first check if our simulation can reproduce the path persistence length reported from experiments. Then, we observe the length of the bent part in the MTs during translocation and find that the length is more extended than that assumed in the theory. This observation resolves the discrepancy between ${L}_{p,path}$ and ${L}_{p,filament}$ of MTs.

## Results

### Microtubule trajectories under external force field

**Figure 2:**
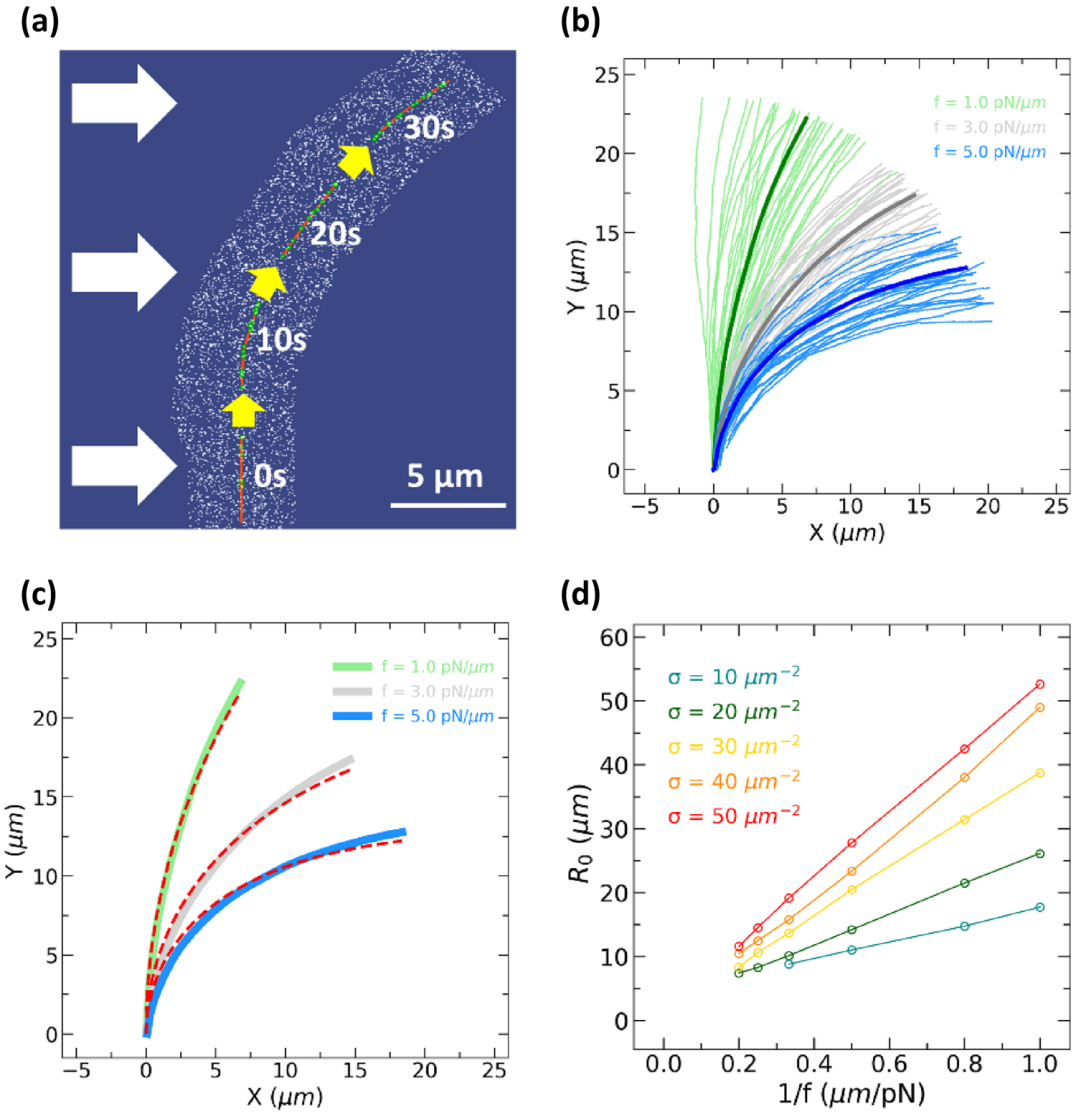
(**a**) A series of snapshots of an MT gliding over kinesin at a uniform external force. The red lines represent the MT; white dots, kinesin; green dots, kinesin binding to the MT. White arrows show the direction of the external force. The kinesin motor density was 30 µm^−2^. Scale bar, 5 µm. (**b**) Paths of MTs under three different external force densities. The green, gray, and blue curves show the 30 representative trajectories of individual MTs for ${f =1.0 \,\,{\text p}{\text N/ \mu} m}$, ${f =3.0 \,\,{\text p}{\text N/ \mu} m}$, and ${f =5.0 \,\,{\text p}{\text N/ \mu} m}$, respectively. The bold curves with corresponding colours show the averages of 30 simulated individual trajectories under the three applied force densities. The kinesin motor density was 30 µm^−2^. (**c**) Averaged path and its radius of curvature. The green, gray, and blue bold curves show the averaged paths of the simulated individual MT trajectories for ${f =1.0 \,\,{\text p}{\text N/ \mu} m}$, ${f =3.0 \,\,{\text p}{\text N/ \mu} m}$, and ${f =5.0 \,\,{\text p}{\text N/ \mu} m}$, respectively. The red broken curves indicate the non-linear fits to the averaged MT trajectories with Eq. (1). The kinesin motor density was 30 µm^−2^. (**d**) The radii of the curvature against the applied force density for various kinesin motor densities ($\sigma$).

Following previous experiments with controlled external force^18,30^, we simulated MT movements under external forces (Fig. 1b). MTs initially moving upward were gradually biased towards the downstream of the applied force density (Fig. 2a, Supplementary Video 1). Above a certain force density depending on the surface motor density (for example, 3.0 pN/µm for 10 µm^−2^), MTs showed occasional dissociations from the kinesin-coated surface, revealed by discontinuities along trajectories. Below the force densities inducing occasional dissociation, MTs followed the trajectories of their leading tips. In this study, we focused only on paths without such occasional dissociations.

For quantitative analysis, 30 MT trajectories under various external forces were simulated. Even under the same applied force density, MT trajectories showed some variance, due to thermal fluctuation (Fig. 2b, Supplementary Video 2). The curvature of MT trajectories became pronounced when increasing the applied force density. To eliminate the variance among MTs and proceed with quantitative analysis, the 30 MT trajectories were averaged. The averaged trajectory at each external force was smooth and had a convex shape, representing progressive alignments of MT trajectories along the applied force fields. The curvature of the averaged trajectory increased with the applied force density (Fig. 2b). The averaged trajectories were compared with the following equation based on the elastic theory of bent rods obtained by van den Heuvel et al.^18^:

1 $$y \left(x\right)= {R}_{0} {cos}^{-1}\left({e}^{-\frac{x}{{R}_{0}}}\right),$$

2 $${R}_{0}= \frac{3{k}_{B}T{L}_{p}}{f{\langle d \rangle}^{2}},$$

where ${k}_{B}$ is Boltzmann constant, $T$ is absolute temperature, $f$ is external force density, $\langle d\rangle$ is the average length of the bent part of MTs. ${L}_{p}$ is either path or filament persistence length as we specify later. The averaged simulated path was fitted with Eq. (1) (Fig. 2c, Fig. S1). There were systematic deviations especially at low motor density and high force density, presumably because of detachments of kinesin motors from MTs by the external force. The radius of the curvature, ${R}_{0}$, was inversely proportional to the applied force density (Fig. 2d). The proportionality depended on the surface motor density.

### Reproduction of experimental path persistence length

**Figure 3:**
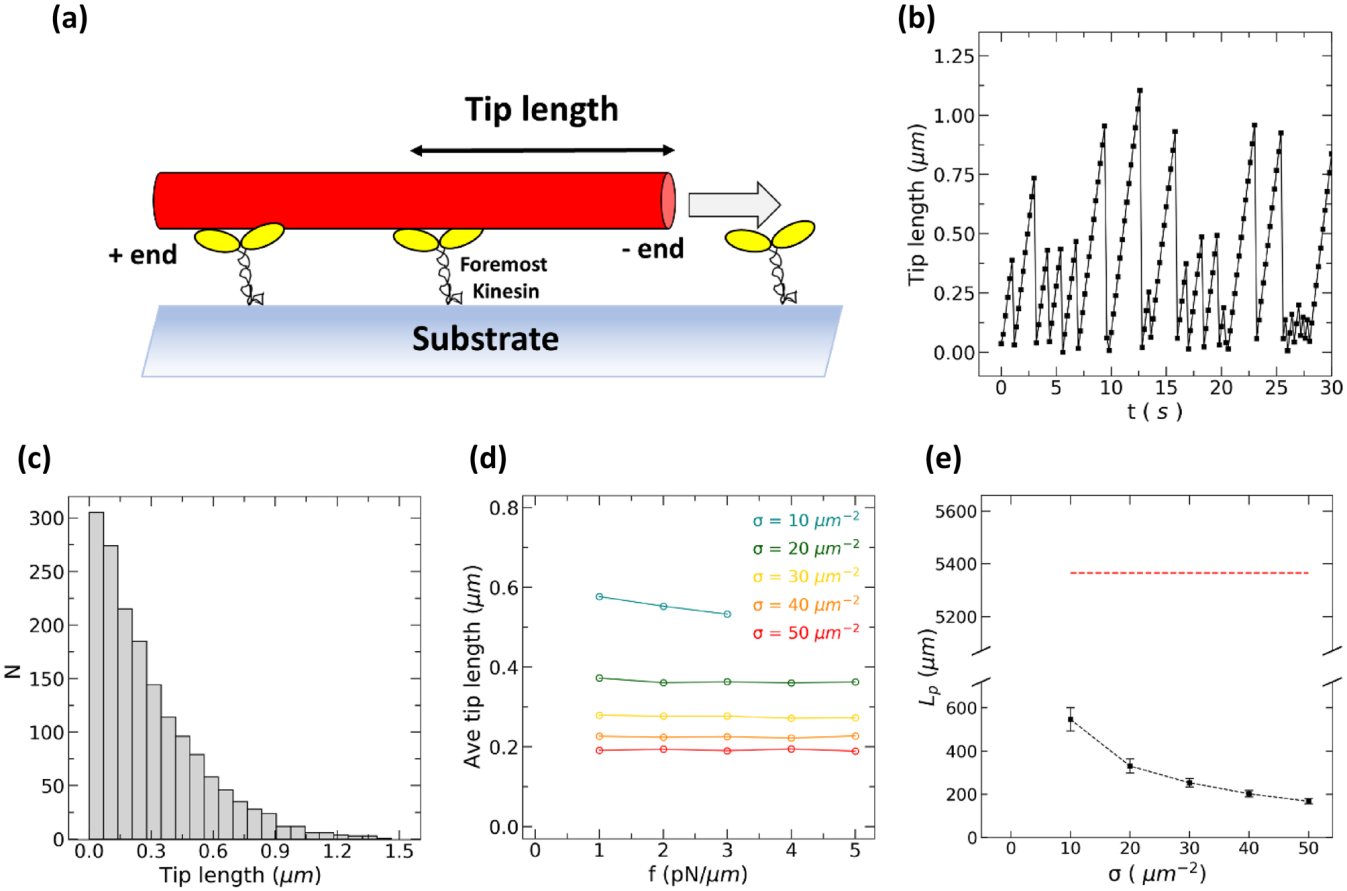
(**a**) A schematic drawing of the tip length. (**b**) Time evolution of the tip length of a gliding MT. (**c**) Frequency distribution of the tip length of a gliding MT. The distribution was Poissonian. The applied force density was ${f =3.0 \,\,{\text p}{\text N/ \mu} m}$. The density of the kinesin motor was 30 µm^−2^. (**d**) The average tip length against the applied force density. The different colours correspond to different motor densities as indicated in the figure. (**e**) The path persistence length as a function of motor density (σ). The red broken line shows the preset value of the filament persistence length. Error bars denote standard deviation.

To check if our simulation reproduced the previous experimental results^18,30^, we calculated ${L}_{p,path}$ from Eq. (2). For this, as ${R}_{0}$ was obtained for various $1/f$ in the previous section, we needed to know the length of the bending part, $d$. By following the theoretical assumption^22^, we took $d$ as the MT tip length between the MT leading end and the foremost kinesin (Fig. 3a). A representative time-evolution of the tip length of a MT is shown in Fig. 3b. The saw-toothed profile was plausible based on the following behaviour. As the foremost kinesin moved upward the MT plus end, the tip length was almost linearly elongated. Upon binding of a new kinesin at the tip, the tip length suddenly shortened. Reflecting random encounters between an MT and kinesins, the distribution of the tip length was an exponential one (Fig. 3c), as considered in a previous study^18^.

We calculated the average tip length of MT with various motor densities, $\sigma$, and applied force density, $f$ (Fig. 3d). The average tip length $\langle d\rangle$ decreased with the increase of the motor density and did not change significantly with the applied force densities.

By substituting the obtained average tip length as $\langle d\rangle$ in Eq. (2), ${L}_{p,path}$ was calculated (Fig. 3e). We found that ${L}_{p,path}$ of MT was around 0.2–0.5 mm, which is much shorter than ${L}_{p,filament}$ of 5 mm that went into the simulation as a fixed parameter (Fig. 3e). ${L}_{p,path}$ obtained here is comparable with the experimentally reported values of 0.1–0.5 mm^16–19^. This simulation result showed that ${L}_{p,path}$ is not necessarily equal to ${L}_{p,filament}$. It should be noted that the discrepancy between ${L}_{p,path}$ and ${L}_{p,filament}$ was reproduced without assuming the length-dependent rigidity of MT^24,25^.

### Length of the fluctuating part in microtubules during translocation

**Figure 4:**
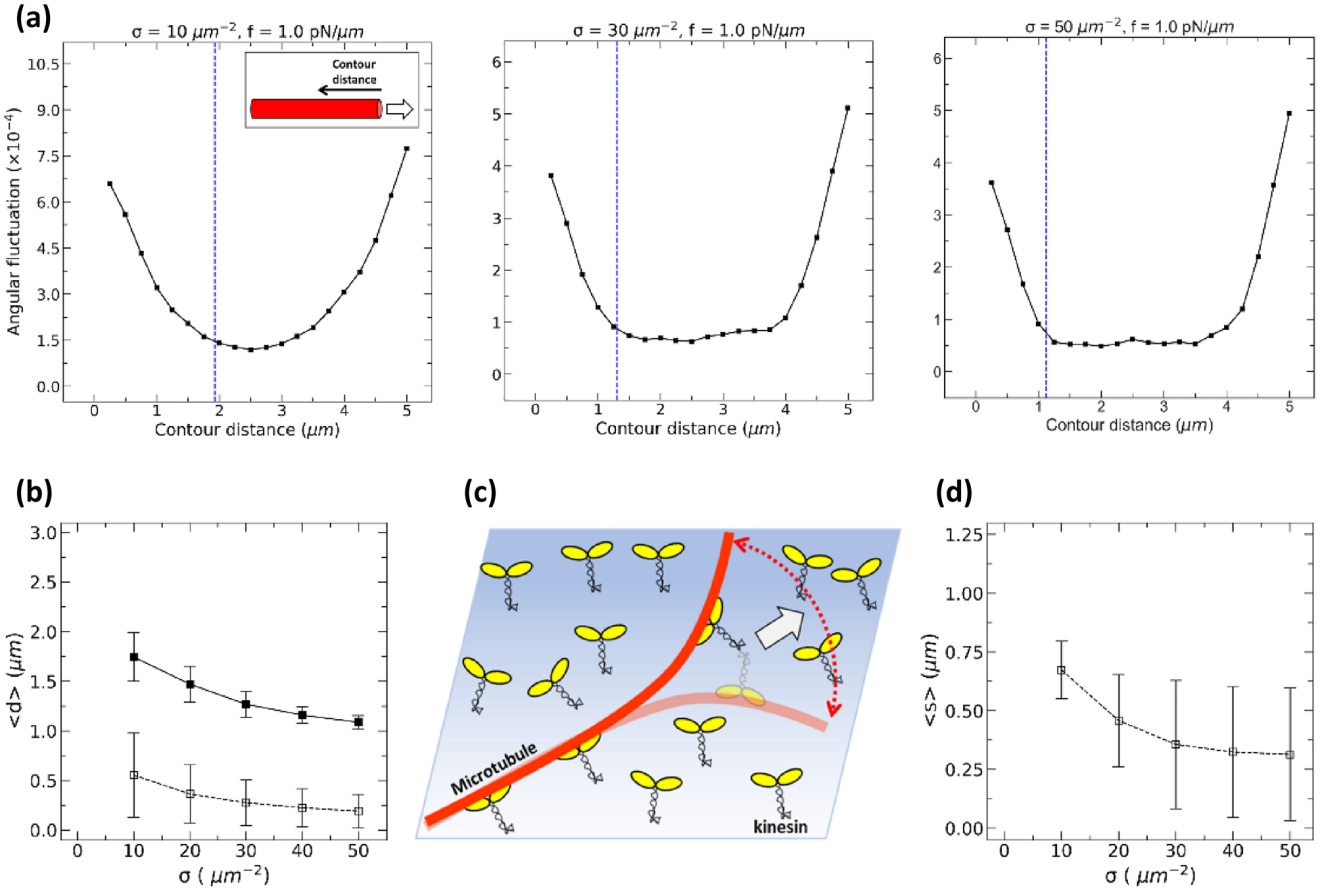
(**a**) Angular fluctuation of MT segments at various locations on the MT denoted by contour distances from their leading tips (minus ends). The solid squares exhibit the angular fluctuation of the MT segment with the motor density ($\sigma$) of 10 µm^−2^ (left), 30 µm^−2^ (middle), and 50 µm^−2^ (right) at ${f =1.0 \,\,{\text p}{\text N/ \mu} m}$ The blue broken line indicates the length of the bent part calculated by using the preset value of ${L}_{p,filament}$ and using the obtained ${R}_{0}$. Inset: definition of contour distance from the MT leading tip. (**b**) Comparison of the length of the bent part and the average tip length. The solid squares represent the length of the bent part, the open squares, the tip length. Error bars denote standard deviation. (**c**) Schematic representation of fluctuation of an MT driven by kinesin motors. (**d**) Averaged spacing along MTs between neighbouring kinesins bound on the MTs. Error bars denote standard deviation.

To test the theoretical assumption that the length of the bending part of MTs is equal to the tip length, we calculated the angular fluctuation of MT segments. Figure 4a shows the variance of angular fluctuations of MT segments as functions of the contour distance from MT leading ends (for details, see Fig. S2). Angular fluctuations were significantly larger at both ends of MTs than those at the central parts of MTs. Since the tip length was 0.25–0.6 µm (Fig. 4b), the length of the part showing significant fluctuation was longer than the tip length, indicating that the theoretical assumption may not hold.

To see if the extended length of fluctuating parts is plausible, we deduced the length of the bending part of MTs by using the preset value of ${L}_{p,filament}$ and using the obtained ${R}_{0}$. By substituting into Eq. (2) the obtained values of ${R}_{0}$ and other preset parameters including ${L}_{p,filament}$ and solving for $\langle d\rangle$, we calculated the length of the bent part to be 1.1–1.7 μm. The calculated length of the bent part was around three times larger than the previously calculated tip length defined (Fig. 4b). The calculated length of the bent part showed good agreement with the range of the fluctuating part obtained by the fluctuation analysis (Fig. 4a).

These simulation results were caused by kinesins being flexible so that the fluctuating part of MT was extended more than the length between MT leading tip and the foremost kinesin as schematically shown in Fig. 4c. Since the average separation between neighboring kinesins on MTs was 0.35–0.67 μm (Fig. 4d; for details, see Supplementary Information), on average, 2–3 kinesins bound to MTs within the bent part of MTs.

## Discussion

In this study, we showed that ${L}_{p,path}$ of gliding MTs over kinesin-coated surfaces could significantly differ from the MT ${L}_{p,filament}$, as opposed to the previous theoretical prediction. This difference arose because the fluctuating part of the MT during its gliding over a kinesin-coated surface extended much longer than the tip length, as previously suggested by Agayan et al.^19^. This happened because the bound kinesins did not act as rigid anchors while the theory assumed that kinesins behave as rigid anchors. Our simulation results showed that the significant difference between ${L}_{p,path}$ and ${L}_{p,filament}$ could arise without assuming the length-dependent MT bending stiffness^24,25^. Thus, the difference between the two persistence lengths can be explained without assuming a length-dependent MT bending stiffness. Measurements of MT ${L}_{p,filament}$ from gliding MT trajectories^17,18^ based on the theoretical prediction that ${L}_{p,path}$ is equal to ${L}_{p,filament}$, may need to be reconsidered.

We showed that ${L}_{p,path}$ resulted from not only the mechanical properties of MT but also the surface density of kinesins. As ${L}_{p,path}$ of gliding MTs over the kinesin-coated surface is a vital parameter for determining the performance of MS applications, the insights obtained here will be useful in designing elements for devices^31^, in reducing error rates in biocomputations^7^, in developing loading channels for MSs^32^, and in optimizing separations of biomolecules^33,34^.

## Simulation method

The simulation method was based on our previous work^35^ which we extended to include external applied force to MTs. In the following, we briefly summarize the simulation method. The trajectory of the MS was modelled as a three-dimensional movement of MTs propelled by kinesin motors.

We assumed the MTs to be infinitely thin and inextensible semiflexible bead-rod polymers with a flexural rigidity of 22.0 pN µm^−2^^36^. The length of MTs was set to be 5 µm and each MT consisted of 20 rigid segments.

Microtubule movement was simulated with Brownian dynamics under the constraint of fixed segment length. In this method, a single time step consisted of the following two steps.

In the first step, the beads representing an MT were moved without considering any constraint, using the following expression:

3 $${r}_{i}^{^{\prime}}\left(t+\Delta t\right)={r}_{i}\left(t\right)+\frac{\Delta t}{\zeta }{F}_{bending,i}+ \frac{\Delta t}{\zeta }{F}_{kinesin,i}+\frac{\Delta t}{\zeta }{F}_{ext}+\sqrt{2D\cdot \Delta t}\cdot {{\varvec{\xi}}}_{{\varvec{i}}},$$

where ${r}_{i}$ is the position vector of the i-th bead consisting of a microtubule, $\zeta$ is the viscous drag coefficient, ${F}_{bending,i}$ is the restoring force of MT bending, ${F}_{kinesin,i}$ is a force exerted by bound kinesin, ${F}_{ext}$ is an external force, $D$ is the diffusion coefficient of the bead, and ${{\varvec{\xi}}}_{i}$ is a three-dimensional random vector whose components take random values with zero mean and standard deviation of one. $\Delta t$ was set at 0.5 × 10^–6^ s to ensure numerical stability. The viscous drag coefficient used was the average of the parallel and perpendicular drag coefficients^37^:

4 $$\zeta =\frac{3\pi \eta d}{\mathrm{ln}\left(\frac{d}{2r}\right)},$$

where $\eta$ is the viscosity of water (0.001 Pa s), $d$ is the length of the MT segment (0.25 µm), and $r$ was the radius of MT (12.5 nm). The diffusion coefficient was calculated using $D={k}_{B}T/\zeta$. We took this length of the MT segmentation such that taking shorter microtubule segmentation lead to negligible change of results (Fig. S4).

The restoring force of MT bending was calculated from the following bending potential^37^:

5 $$U=\frac{1}{2} \frac{EI}{{d}^{3}} \sum_{i=2}^{n-1}{\left({r}_{i+1}-2{r}_{i}+ {r}_{i-1}\right)}^{2},$$

where $EI$ is the flexural rigidity.

Kinesin motors were randomly distributed over the allowed surface by specifying the positions of the kinesin tails (Fig. 1b). If an MT segment came close to a kinesin motor tail within a capture radius (20 nm)^22^, the kinesin motor was assumed to be bound to the MT segment, and the position of the motor head was specified on the MT segment. Once bound, the head of the bound kinesin motor moved toward the MT plus end with a force-dependent velocity expressed as

6 $$v\left({F}_{\| }\right)={v}_{0}\left(1-\frac{{F}_{\| }}{{F}_{stall}}\right),$$

where ${v}_{0}$ is the translational velocity without applied force, ${F}_{\| }$ is the component of the pulling force along the MT, and ${F}_{stall}$ is the stall force of the kinesin motors. ${v}_{0}$ was set at 0.8 µm/s, and ${F}_{stall}$ was set at 5 pN.

The bound kinesin acted as a linear spring between the motor head and tail with the spring constant of 100 pN/µm^38^ and with an equilibrium length of zero and exerted a pulling force on the MT segment. The pulling force was divided into two forces which acted on the two beads located at either end of the MT segment where the kinesin motor was bound, under the condition that the total force and torque on the segment remained the same.

A kinesin motor bound to an MT detached when tension reached 7 pN. By following the approach taken by Gibbons et al.^39^, we neglected the spontaneous dissociation of the bound kinesin from the MT.

As the external force, a uniform force field pointing in the x-direction was applied:

7 $${F}_{ext} =\left(\begin{array}{c}f\cdot d\\ 0\\ 0\end{array}\right),$$

where, $f$ is a force density and ${F}_{ext}$ is an external force.

In dealing with Eq. (3), we used an implicit–explicit method, where the restoring force of MT bending was implicitly calculated while other terms were explicitly calculated.

In the second step, the unconstrained movements were corrected by considering the constraints due to the segment length and the guiding tracks. To keep the segment length constant, the coordinates of the beads representing the MT ${\{r}_{i}\}$ as shown were subject to the following holonomic constraints:

8 $${g}_{segment,k}={\left({r}_{k+1}-{r}_{k}\right)}^{2}-{d}^{2}=0 \left(k=1, \dots , n-1\right).$$

To keep the MT movement above the substrate, the position of the beads representing the MT were subjected to the following holonomic constrains:

9 $${g}_{track,i}= {z}_{i}=0, if {z}_{i}<0.$$

The correction was carried out with the following expression:

10 $${r}_{i}\left(t+\Delta t\right)={r}_{i}^{^{\prime}}\left(t+\Delta t\right)+ {\Delta r}_{i}\left(t+\Delta t\right),$$

where ${\Delta r}_{i}(t+\Delta t)$ is the correction term

11 $${\Delta r}_{i}\left(t+\Delta t\right)=\frac{\Delta t}{\zeta }\sum_{k=1}^{n-1}{\lambda }_{segment,k}\frac{{\partial g}_{segment,k}}{{\partial r}_{i}}+\frac{\Delta t}{\zeta }{\lambda }_{track,i}\frac{{\partial g}_{track,i}}{{\partial r}_{i}},$$

and ${\lambda }_{segment,k}$ and ${\lambda }_{track,i}$ are Lagrangian multipliers, which were determined in order for the coordinate at $t+\Delta t$ to satisfy the constraints given by Eqs. (8) and (9), respectively. For this, we went through the calculations for the constraints one by one, cyclically, adjusting the coordinates until the constraints were satisfied with a tolerance of 10^–6^ µm.

Simulation results were visualized with ParaView (https://www.paraview.org/).

## Supplementary Information

Supplementary Information.

Supplementary Video 1.

Supplementary Video 2.

## Data Availability

The datasets generated during and/or analysed during the current study are available from the corresponding author on reasonable request.
