# Supplementary material for: Linking path and filament persistence lengths of microtubules gliding over kinesin
Source: Sci Rep. 2022 Feb 23;12:3081. doi: 10.1038/s41598-022-06941-x (PMC8866476; doi:10.1038/s41598-022-06941-x)
Supplement: Supplementary file 1 — Supplementary Information. [file 41598_2022_6941_MOESM1_ESM.docx]

**Supplementary Information**

**Linking path and filament persistence lengths of microtubules gliding over kinesin**

May Sweet, Samuel Macharia Kang’iri and Takahiro Nitta*

Applied Physics Course, Faculty of Engineering, Gifu University 501-1193 Japan

**The averaged path of the microtubule and its radius of curvature**


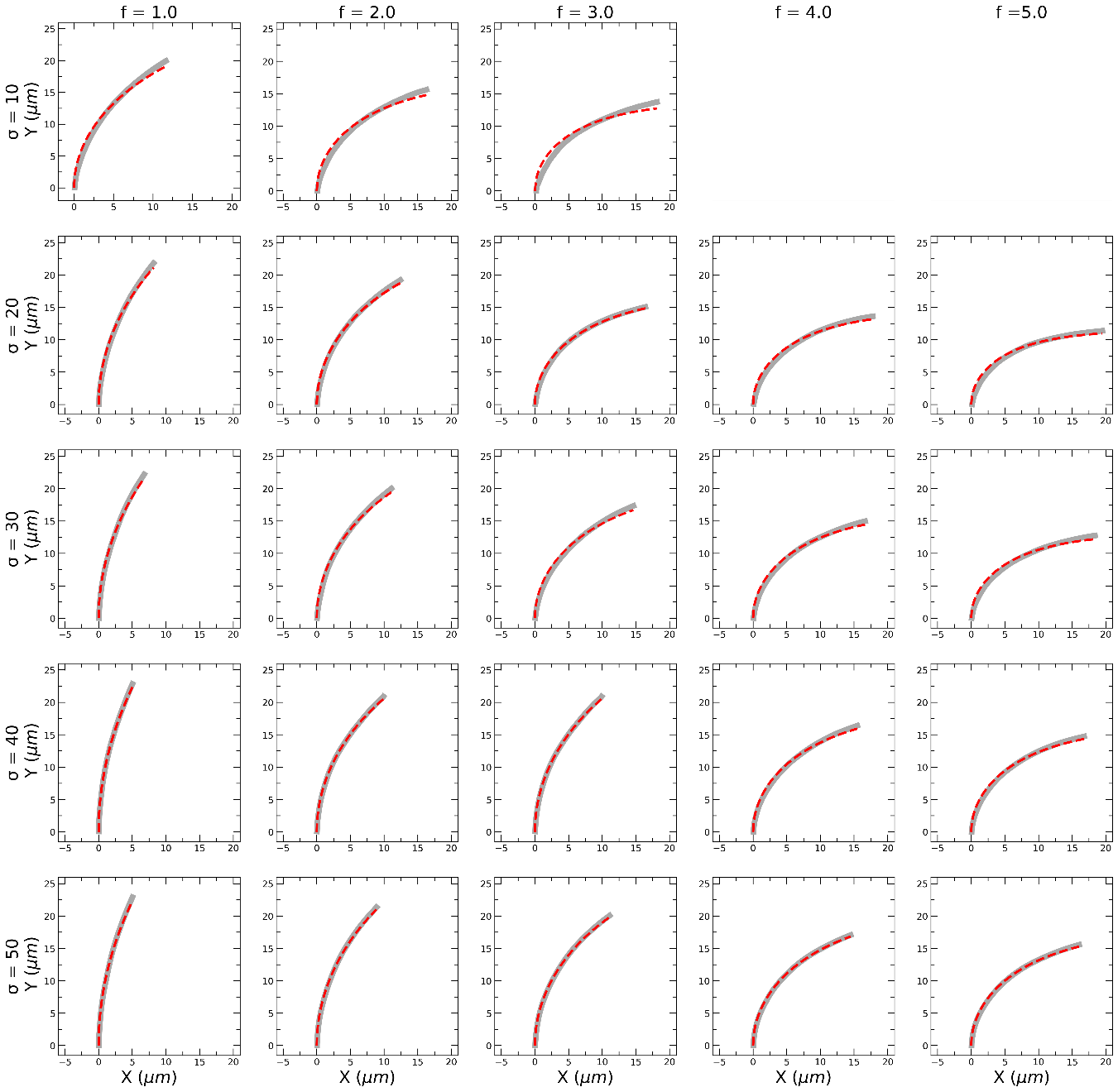


Figure S1. Averaged path and its radius of curvature of microtubules with the motor density of 10 µm^-2^ (the first row), 20 µm^-2^ (the second row), 30 µm^-2^ (the third row), 40 µm^-2^ (the fourth row) and 50 µm^-2^ (the fifth row) at various external force densities (f = 1.0 pN/µm, 2.0 pN/µm, 3.0 pN/µm, 4.0 pN/µm and 5.0 pN/µm). The grey bold curves show the averaged paths of simulated microtubule trajectories. The red dotted curves indicate the non-linear fits to the averaged microtubule trajectory with equation (1) in main manuscript.

**Angular fluctuation of microtubule segments**

**
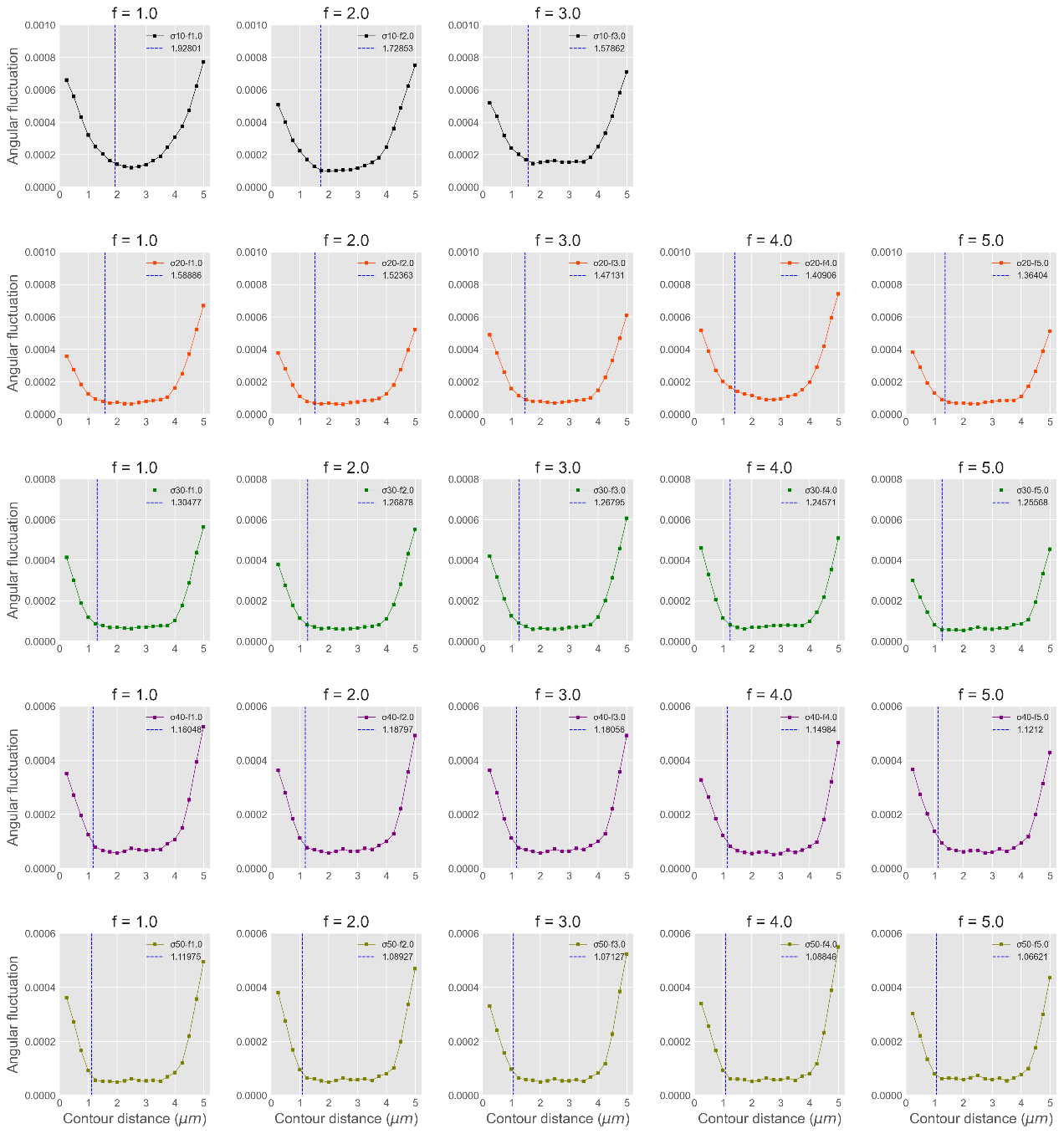
**

Figure S2. Angular fluctuation of microtubule segments at various locations denoted by contour distances from their leading tips (minus ends). The solid lines with solid squares exhibit the angular fluctuating of the MT segment with the motor density of 10 µm^-2^ (the first row), 20 µm^-2^ (the second row), 30 µm^-2^ (the third row), 40 µm^-2^ (the fourth row) and 50 µm^-2^ (the fifth row) at various external force densities (f = 1.0 pN/µm, 2.0 pN/µm, 3.0 pN/µm, 4.0 pN/µm and 5.0 pN/µm). The blue dashed lines show the calculated length of the bent part of microtubules.

**The tip length and spacing** **between binding kinesins on microtubules**

Here we discuss the tip length (Fig. 3A) and spacing between binding kinesins on microtubules. Estimates on the tip length and the spacing have been previously discussed [1] [2], as schematically shown in Fig. S3(A). The average tip length, $\left\langle d \right\rangle$, and spacing, $\left\langle s \right\rangle$, can be calculated to be:

$$\left\langle s \right\rangle=\frac{L}{2\sigma Lw},$$

$$\left\langle d \right\rangle=\frac{1}{2}\left\langle s \right\rangle,$$

where $\sigma$ is the motor density on the substrate and $L$ is the length of a microtubule. The lower limit of $w$ can be given by the capture radius of kinesin motors, 20 nm, leading to the upper limit of the estimate of $\left\langle s \right\rangle$ and $\left\langle d \right\rangle$ represented by the blue line in Figs. S3(B, C). The upper limit of $w$ can be given by ${F_{d}}/k$, which is the maximum extension of kinesins since microtubules show lateral fluctuation so that $w$ can be wider than the capture radius of kinesin motors. With $w={F_{d}}/k$, the estimated $\left\langle s \right\rangle$ and $\left\langle d \right\rangle$ can be obtained as represented by the red line in Figs. S3(B, C). The $\left\langle d \right\rangle$ and $\left\langle s \right\rangle$ obtained from the simulation fell within this range.


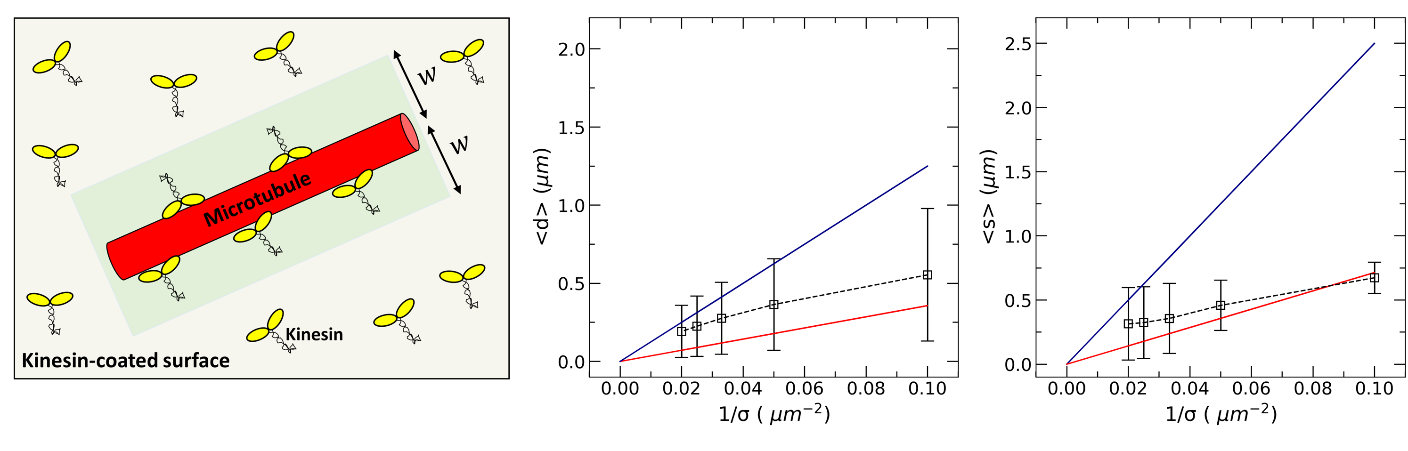
(A) (B) (C)

Figure S3. (A) Schematics of estimates of the tip length and spacing between binding kinesins on microtubules. (B) The average tip length of microtubules against the inverse of the motor density. The blue and red lines represent the upper and lower estimates described above, respectively. (C) The average spacing between binding kinesins on microtubules against the inverse of the motor density. The blue and red lines represent the upper and lower estimates described above, respectively.


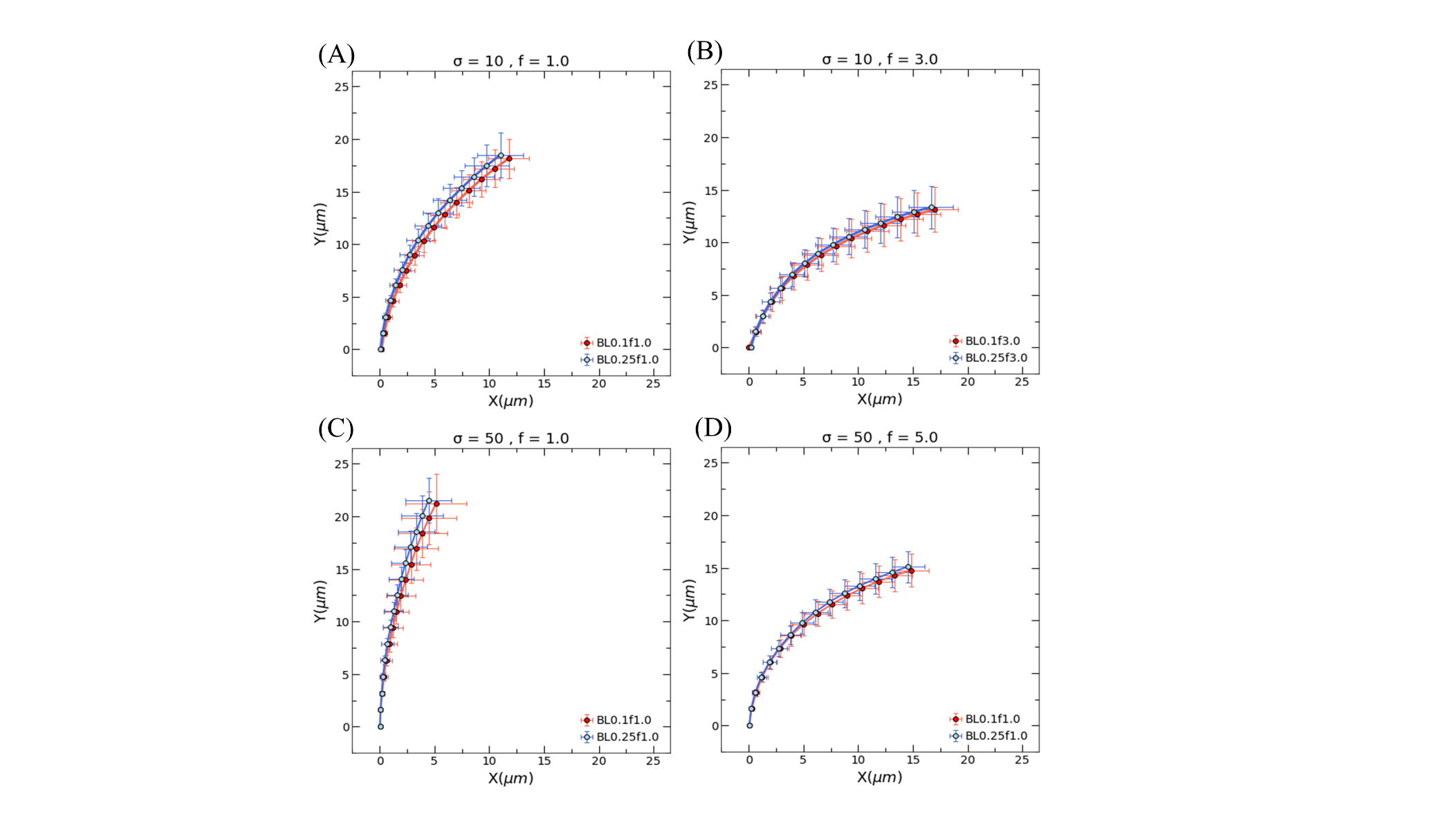
**Effect of segmentation length of microtubule**

Figure S4 Comparison of averaged paths of MTs with 0.1 µm (red marks) and 0.25 µm (blue marks) segmentation length. The error bars denote standard deviation. (A, B) The kinesin motor density is 10 µm^-2^ (the lowest motor density). (A) The external force density is 1.0 $pN/\mu m$ (the lowest force density). (B) The external force density is 3.0 $pN/\mu m$ (the highest force density for the motor density of 10 µm^-2^). (C, D) The kinesin motor density is 50 µm^-2^ (the highest motor density). (C) The external force density is 1.0 $pN/\mu m$ (the lowest force density). (D) The external force density is 5.0 $pN/\mu m$ (the highest force density).

**Supplementary Videos (Caption)**

**Supplementary Video 1 | A microtubule gliding over kinesin at a uniform external force directing toward the right.** The surface density of kinesin was 30 µm^-2^. The orange line represents an MT under the external force of $3.0 pN/\mu m$. The white dots represent the kinesin motors, and the green dots represent the kinesin binding to the MT. The white arrows indicate the direction of the external force.

**Supplementary Video 2 | Gliding movements of microtubules under external forces directing toward the right.** The surface density of kinesin was 30 µm^-2^. The yellow and orange curves showed the 10 representative trajectories of individual MTs under the two different external force densities of $1.0 pN/\mu m$ and $3.0 pN/\mu m$, respectively. The gliding movements were smooth, and the MTs followed their leading tips. The trajectories gradually aligned themselves to the direction of the external forces. Yellow and orange arrows on the left indicate the direction of the external force densities of 1.0 pN/$\mu$m and 3.0 pN/$\mu$m respectively.

**References**

[1] T. Duke, T. E. Holy, and S. Leibler. Gliding Assays’ for Motor Proteins: A Theoretical Analysis. *Phys. Rev. Lett.* **74**, **2**, 330–333. doi: 10.1103/PhysRevLett.74.330 (1995).

[2] T. Q. P. Uyeda, S. J. Kron, and J. A. Spudich. Myosin step size. Estimation from slow sliding movement of actin over low densities of heavy meromyosin. *J. Mol. Biol.* **214**. **3**. 699–710. doi: 10.1016/0022-2836(90)90287-V (1990).
